# Visualization of the chloroplast MinE ring in living mesophyll cells of *Arabidopsis thaliana*

**DOI:** 10.1080/15592324.2026.2686341

**Published:** 2026-06-24

**Authors:** Makoto T. Fujiwara, Ryuuichi D. Itoh

**Affiliations:** a Department of Materials and Life Sciences, Faculty of Science and Technology, Sophia University, Chiyoda, Japan; b Department of Chemistry, Biology and Marine Science, Faculty of Science, University of the Ryukyus, Okinawa, Japan

**Keywords:** AtMinE1, chloroplast division, FtsZ, Min system, plastid division

## Abstract

Chloroplast division is a complex process influenced by various proteins, among which bacteria-derived MinE plays a vital role in correctly placing the FtsZ-based division apparatus and initiating the division. This study aimed to elucidate the intrachloroplastic localization of MinE in the living mesophyll cells of *Arabidopsis thaliana* using the MinE-yellow fluorescent protein (YFP) fusion. Fluorescence microscopy of the complemented *A. thaliana minE* mutant, expressing *MinE-YFP* under the native *MinE* promoter, showed normal chloroplast division and revealed a mid-chloroplast MinE ring composed of array-of-dots and filaments, as well as other distinct localization patterns of MinE during chloroplast division. The MinE ring relatively maintained its configuration during chloroplast constriction, as does the FtsZ ring, suggesting a dynamic protein dissociation correlating with division progression. We also observed a unique “twin dot” structure and one-sided distribution of MinE in chloroplasts with slight over-accumulation of MinE-YFP in transgenic tissues. These observations deviate from the well-accepted model of the MinE behavior during *Escherichia coli* cytokinesis, suggesting fundamental differences in the division mechanisms between chloroplasts and bacteria. The present findings deepen our understanding of the protein organization at the chloroplast division site and highlight the distinctive nature of the MinE protein’s behavior in plants.

## Introduction

Modern chloroplasts and cyanobacteria share common protein components for their replication by binary fission.^1^ Among them, FtsZ plays a crucial role in the cell/organelle division machinery. FtsZ is a tubulin-related GTPase, which assembles into a ring structure, the so-called Z-ring, beneath the plasma membrane on the cytoplasmic side in most bacteria and some groups of archaea. The Z-ring is usually formed at the mid-cell division site and serves both as the scaffold for other enzymes required for septum formation and as the generator of contractile force for membrane invagination. In chloroplasts of eukaryotic algae and land plants, FtsZ is encoded in the nuclear genome and is synthesized by cytoplasmic ribosomes before being imported into the chloroplast stroma (corresponding to the cytoplasm of prokaryotes). In the stroma, the Z-ring is formed at the mid-chloroplast division site beneath the inner envelope membrane (equivalent to the plasma membrane of prokaryotes). Chloroplasts have a double envelope-membrane structure, which requires the coordinated invagination of the outer and inner envelope membranes and the constriction and pinching-off of the outer envelope membrane.^2^ ^,^ ^3^ Studies on chloroplast division in mesophyll cells of *Arabidopsis thaliana* have identified “eukaryote/chloroplast-specific” components such as the dynamin-related protein DRP5B (ARC5) and the PDV1 and PDV2 proteins which localize at the outer envelope membrane.^4^ ^,^ ^5^

In addition to FtsZ, division-site regulator proteins MinD and MinE are conserved in chloroplasts of the green lineage and in bacteria, including cyanobacteria.^6^ ^,^ ^7^ Studies of cell division in the bacterium *Escherichia coli* led to the “Min system” model for division-site determination.^8^ ^,^ ^9^ MinD, in cooperation with another Min protein, MinC, inhibits the polymerization of FtsZ and thereby prevents Z-ring formation. MinE assembles into a membrane-bound ring-like structure (E-ring), which oscillates from cell pole to cell pole. The oscillatory movement of the MinE ring clears MinCD inhibitory complexes from the midcell to both cell poles and thereby establishes a MinCD-free central region, where FtsZ assembles into the Z-ring. The MinE-driven, pole-to-pole oscillation of MinC and MinD was also demonstrated in the cyanobacterium *Synechococcus elongatus* PCC 7942,^10^ although the presence of a MinE ring in cyanobacteria has not, to our knowledge, been reported. The bacterial Min system model was originally proposed based on molecular genetic analyses of cell division in *E. coli.* ^11^ ^,^ ^12^ These analyses demonstrated that loss of MinE function (or overproduction of MinD) inhibits division initiation, whereas loss of MinD function (or overproduction of MinE) results in uncontrolled, asymmetric, or multiple divisions that produces “minicells” (the origin of the gene prefix “*min*”). Similar results have been observed in chloroplast division in the leaf mesophyll (cortex) cells of overexpression lines and or loss-of-function mutants of *A. thaliana MinD* and *MinE*, as demonstrated by our group and others.^13-20^ However, there are important differences between chloroplasts and bacteria. In plants, both MinD and MinE are nuclear-encoded, like FtsZ.^13^ ^,^ ^21^ In addition, no apparent homolog of the *minC* gene has been identified in plants or algae to date.^22^

While the molecular mechanism of mesophyll chloroplast division has been extensively studied, our knowledge of the suborganellar localization and distribution patterns of chloroplast Min proteins remains limited. This limitation hampers evaluation of whether the *E. coli* Min system model applies to chloroplast division. Using immunofluorescence staining, studies have shown that MinD (AtMinD1) localizes to the mid-chloroplast division site and to punctate structures within chloroplasts in *A. thaliana.* ^23^ MinE (AtMinE1) was later found to colocalize with MinD and to form a ring-like structure in dividing chloroplasts of *A. thaliana.* ^24^ Our group also confirmed that a functional, double hemagglutinin epitope-tagged MinD (MinD-dHA) showed a localization pattern similar to that of the native protein in chloroplasts of complemented *minD* (*arc11*) plants.^25^ While the presence of mid-chloroplast MinE rings is partly consistent with the *E. coli* Min system model,^26^ colocalization of MinD and MinE at the chloroplast division plane indicates fundamental differences in the Min system between chloroplasts and *E. coli* cells. Because the above observations were carried out using fixed mesophyll cells as materials, information on the spatiotemporal changes in Min protein localization could not be obtained from these analyses. Imaging of fluorescent protein (FP)-tagged proteins in living cells is a powerful approach that complements immunolocalization techniques. Expression of FP-tagged proteins driven by their native promoters that complement mutant phenotypes *in planta* is considered the most reliable strategy. This is because chloroplast division is highly sensitive to the expression levels of division regulators. Imbalance in the MinD/MinE ratio, caused by over- or underexpression of FP-tagged proteins, can lead to division arrest, unequal division, or multiple fission of chloroplasts. Such defects would prevent accurate observation of Min protein localization in properly dividing chloroplasts (*i.e.*, undergoing binary and equal fission).^15^ ^,^ ^25^ A similar issue likely applies to the analysis of PARC6, another chloroplast division site regulator.^27^ ^,^ ^28^

In the present study, we used transgenic lines in the *A. thaliana minE* mutant background that express a functional MinE-yellow fluorescent protein (YFP) under the native promoter of the *A. thaliana MinE* gene.^29^ We carefully selected plants in which chloroplasts divide normally and in which overexpression effects of MinE-YFP were minimized. Although our recent study reported live imaging of a plastid MinE ring in dividing amyloplasts of *A. thaliana* ovule integuments,^30^ live visualization of the MinE localization in chloroplasts has not previously been achieved. Therefore, using these transgenic plants, we conducted imaging of chloroplast MinE in living leaf mesophyll cells and identified a mid-chloroplast ring of MinE as well as additional distinct localization patterns of MinE in dividing chloroplasts.

## Materials and methods

### Plant materials and growth conditions

The *Arabidopsis thaliana minE* mutant (FLAG_056G07, Ws background; Samson et al.^31^) carrying a T-DNA insertion in intron 1 of *MinE* (*atminE1-1* allele; Fujiwara et al.^14^) was obtained from the Institut National de la Recherché Agronomique (INRA; Versailles, France). A complemented *minE* line, E1v24, in which a full-length MinE-YFP fusion gene was expressed under the native *MinE* promoter (*MinE*p:*MinE-YFP*) was previously generated.^29^ A fluorescent *minE* line (*minE* × FC1-7), expressing plastid-targeted CFP under the constitutive CaMV*35S* promoter in the *minE* background, was generated by crossing ♀*minE* plants with ♂FC1-7 plants.^29^ Another fluorescent line (E1v24 × FC1-7), expressing plastid-targeted CFP in the E1v24 background, was generated by crossing ♀E1v24 plants with ♂*minE* × FC1-7 plants. Complementation of chloroplast division defects in the E1v24 line was achieved in plants heterozygous for the T-DNA insertion carrying *MinE*p:*MinE-YFP*.

Seeds were surface-sterilized and grown on agar-solidified Murashige and Skoog (MS) medium under long-day light conditions (16 h light/8h dark) as previously described.^29^

### Microscopy

To confirm expression of stroma-targeted fluorescent proteins in transgenic *Arabidopsis* lines, fluorescence stereomicroscopy was performed with a Leica MZ10 F microscope (Leica Microsystems, Heidelberg, Germany) equipped with filter sets for CFP and YFP (Leica).

To observe living cells and chloroplasts in *Arabidopsis* leaves, fluorescence microscopy was performed with an Olympus IX71 inverted microscope (Olympus, Tokyo, Japan) equipped with emission filters for YFP (FF01-545/55) (Semrock, Rochester, NY, USA), CFP (FF01-483/32) (Semrock), and chlorophyll (BA575IF) (Olympus), using a 40 × objective lens (NA 1.25; UPLSAPO40XS). Samples were prepared as described previously.^28^ Specimens were illuminated using a mercury lamp (U-HGLGPS, Olympus), and fluorescence signals were captured with a Hamamatsu ORCA-Flash2.8 CMOS camera (Hamamatsu Photonics, Hamamatsu, Japan). Bright-field images were obtained with differential interference contrast (DIC) optics. Images were processed using ImageJ 1.43j (http://rsb.info.nih.gov/ij/) and Adobe Photoshop CS6 (Adobe, San Jose, CA, USA).

## Results and discussion

**Figure 1.**
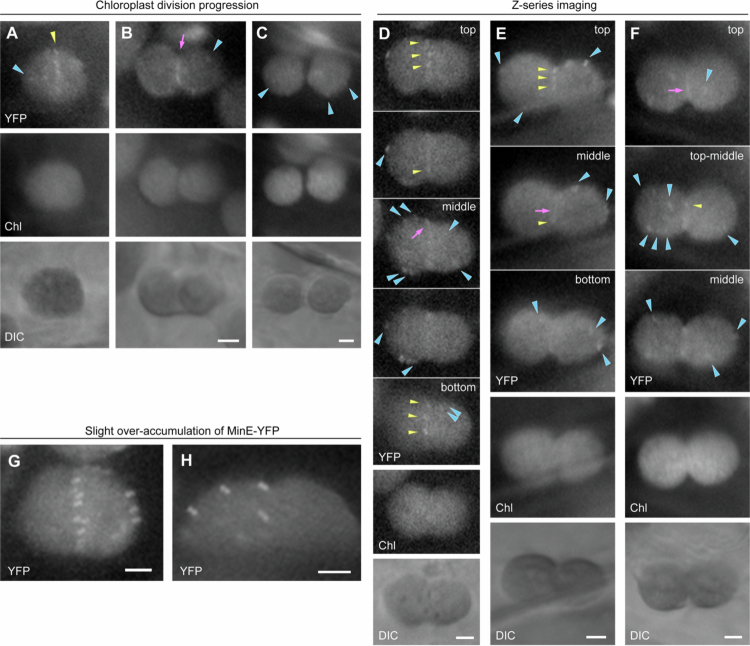
Localization of MinE-YFP in leaf mesophyll cells of *Arabidopsis*. (A–F) Images of chloroplasts in leaf petioles of 15–19-day-old seedlings of complemented *minE* plants. Images of full-length MinE-YFP (YFP), chlorophyll (chl), and DIC are shown. In (D–F), z-series images of MinE-YFP are shown. Yellow and cyan arrowheads indicate punctate signals of MinE-YFP at the mid-chloroplast region and at other regions of the chloroplast, respectively. Arrows indicate filamentous signals of MinE-YFP. (G, H) Twin-dot structures of MinE-YFP in chloroplasts exhibiting slight over-accumulation of MinE-YFP in transgenic tissues. Scale bars: 2 µm.

We investigated the suborganellar localization of MinE in the MinE-YFP-expressing complemented *minE* line (E1v24; Fujiwara et al.^29^). The petiole mesophyll (cortex) cells of this line contained normally sized and shaped chloroplasts (Figure 1A). Some chloroplasts appeared to be undergoing (Figure 1B) or had just completed (Figure 1C) binary and equal division, similar to those in wild-type plants, as judged from chlorophyll autofluorescence (Chl) and DIC images. This phenotype suggests that the expression level of the *MinE-YFP* transgene in this line is within an appropriate range to rescue the dysfunction of the endogenous *MinE* gene. In round or ovoid chloroplasts, which appeared not to have initiated division, a portion of the MinE-YFP proteins preferentially localized to the mid-chloroplast region, likely representing the future division plane, in an array-of-dots pattern (Figure 1A). Besides the central MinE ring, punctate signals within diffuse MinE-YFP signals were also observed. Preferential localization of MinE at the division plane became more evident in constricted chloroplasts undergoing division (Figure 1B). The annular nature of the mid-chloroplast-concentrated MinE localization was confirmed by serial images of individual chloroplasts taken at different focal planes (Z-series imaging; Figure 1D–F). Intriguingly, in these images, we found that two types of signal patterns of MinE-YFP constitute the MinE ring: array-of-dots (yellow arrowheads) and continuous filaments (magenta arrowheads). As for the array-of-dots pattern, the intervals between dots appeared to be irregular (compare yellow arrowheads in Figure 1D and E). MinE also still localized to the periphery of dividing chloroplasts as isolated patches or distinct “twin dots” (see below for details), exhibiting no apparent spatial regularity (cyan arrowheads; Figure 1D–F). The *E. coli* Min system model predicts that MinE would localize asymmetrically to one side of the division plane, but this was not the case for chloroplast MinE. This indicates fundamental differences in the Min system between bacteria (particularly *E. coli*) and chloroplasts.

The localization patterns of MinE-YFP were reproducibly observed in at least four independent experiments. Examination of the fluorescence images showed that, among 79 normally sized and shaped chloroplasts, 45 exhibited dotted MinE-YFP signals, whereas 47 exhibited filamentous signals. Because both dotted and filamentous patterns could coexist within a single chloroplast (*e.g.* Figure 1D–F), the total number exceeds the number of chloroplasts examined. Notably, slight differences in focal planes occasionally caused the MinE-YFP signals to appear predominantly dotted or filamentous, whereas in favorable cases both patterns could be visualized within the same chloroplast (*e.g.* Figure 1D–F). These observations suggest that dotted and filamentous MinE structures may frequently coexist within a single chloroplast. It is also possible that these two structural patterns are reversibly interconvertible, potentially through local nucleation/dissociation processes.

FtsZ and PARC6, like MinE, are chloroplast division regulator proteins and, during chloroplast division, concentrate at the division plane to form ring-like structures.^27^ ^,^ ^28^ ^,^ ^32-34^ In our previous studies, we observed that, as chloroplast division progressed, the fluorescence intensity of FP-tagged FtsZ remained largely constant, while that of PARC6 increases at the medial constricting region,^14^ ^,^ ^28^ suggesting that these rings undergo distinct constriction mechanisms. In this respect, the chloroplast MinE ring resembled the FtsZ ring rather than the PARC6 ring: the signal intensity of MinE-YFP did not appear to correlate with the progression of chloroplast division (Figure 1A, B, D–F). This suggests that MinE proteins gradually dissociate from the ring, in parallel with the decreasing diameter of the constricted site of dividing chloroplasts. This hypothesis may explain the disappearance of ring-like structures of MinE between paired chloroplasts that appear to be immediately post-division, whereas dotted signals in the daughter chloroplast bodies were still discernible (Figure 1C). This contrasts with PARC6, which has been reported to persist at one of the chloroplast poles as a single spot even after the separation of daughter chloroplasts.^27^

In leaf tissues of our *minE* transgenics, we could occasionally find chloroplasts that appeared to exhibit slight over-accumulation of MinE-YFP as indicated by a slight increase in chloroplast size and the fluorescence intensity of YFP (Figure 1G, H). These chloroplasts exhibited a single constriction at the mid-chloroplast region, indicating that they still maintained the ability to undergo symmetric binary fission. MinE-YFP localized to both the mid-chloroplast region and the chloroplast periphery in these chloroplasts. Interestingly, in these chloroplasts, the MinE-YFP signal was clearly observed as a pair of dots (“twin dots”). The twin dots could be unusually arranged at the mid-chloroplast. In addition, the peripheral twin dots were detected only on one side of the dividing chloroplasts. Upon careful observation, the existence of such twin dots could be confirmed in fully complemented chloroplasts (Figure 1D, E). The one-sided distribution of MinE in the slightly protein over-accumulated chloroplasts is reminiscent of the behavior of MinE (and MinCD) during the division of *E. coli* cells. However, due to rapid photobleaching of the relatively weak MinE-YFP signal during excitation, the emergence and fate of this MinE distribution could not be determined. At present, there is no evidence for the intraorganellar pole-to-pole oscillation of MinE in dividing chloroplasts, although the data may indicate that the chloroplast MinE has the potential to form twin dot structures and to adopt a one-sided (asymmetric) distribution within the stroma. In particular, the asymmetric distribution of MinE cannot be explained by MinE alone but must be produced by its interaction with other stromal or inner-envelope components. These observations suggest the presence of a chloroplast factor that shows asymmetric distribution in the natural state. This factor may be a protein such as MinD or a specific lipid species.

In this context, previous studies demonstrated physical interactions among chloroplast Min-system components, including MinD, MinE, and ARC3, which is thought to functionally substitute for bacterial MinC,^35-37^ as well as colocalization of MinD and MinE in dividing chloroplasts.^24^ However, whether the chloroplast MinE ring directly associates with the FtsZ ring or how these Min-system components are coordinately organized in living plastids remains unresolved. Further live-cell imaging and molecular interaction analyses will be required to clarify the spatial and functional relationship between the chloroplast Min system and the FtsZ division machinery.

Chloroplasts differentiate from proplastids in higher plant tissues, and the function of Min proteins in relation to plant growth and development is also a subject of our study.^38^ ^,^ ^39^ Our recent study identified a MinE ring in dividing amyloplasts of ovule integuments in *A. thaliana.* ^30^ While the *minE* mutation broadly affects plastid morphology across multiple tissues,^38^ the plastid phenotype in the mutant varies among leaf cell types,^29^ suggesting that division mechanisms differ among plastid types. Despite this variability, our results indicate that plastid MinE consistently forms a ring structure at the putative division plane in different plastid types (at least mesophyll chloroplasts and integument amyloplasts). Thus, our present observations suggest a broadly conserved role of the MinE ring in plastid division.
